# Breastfeeding Rate, Food Supplementation, Dietary Diversity Among Children Aged 6–59 Months, and Associated Factors in Papua New Guinea

**DOI:** 10.3389/fnut.2021.622645

**Published:** 2021-02-19

**Authors:** Bang Nguyen Pham, Vinson D. Silas, Anthony D. Okely, William Pomat

**Affiliations:** ^1^Population Health and Demography Unit, Papua New Guinea Institute of Medical Research, Goroka, Papua New Guinea; ^2^School of Health & Society and Early Start, University of Wollongong, Illawarra Health and Medical Research Institute, Wollongong, NSW, Australia

**Keywords:** food supplementary, dietary diversity, CHESS, Papua New Guinea, breastfeeding

## Abstract

**Background:** Along the socioeconomic changes in the past decades, Papua New Guinea (PNG) has undergone significant food transition. Little is known about the influence of household and maternal socioeconomic demographic factors on dietary intake and diversity among children under 5 years of age (CU5).

**Objective:** This study aimed to examine breastfeeding rate, food supplementation, dietary intake, and diversity among children aged 6–59 months and to identify associations with household and maternal socioeconomic demographic factors in PNG.

**Method:** Data from 2,943 children were extracted from the Comprehensive Health and Epidemiological Surveillance System database, operated by the PNG Institute of Medical Research and used to estimate breastfeeding rate, food supplementation, and dietary intake of CU5 in a typical week. Dietary diversity score (DDS) was used as a proxy indicator to measure nutrient adequacy. Associations of DDS with household and maternal socioeconomic and demographic factors were examined using multivariate logistic regression analysis.

**Result:** The breastfeeding rate among children aged 6–8 months was 85% (70% in urban and 90% in rural sectors), and 50% of children of this age group were fed with supplementary foods. Twenty percent of children aged 6–23 months were currently breastfed and received solid, semisolid, and soft foods three times or more per day. Forty-eight percent of children aged 6–59 months had a total DDS below the average level (23 scores). Place of residence, mother's education, and household wealth were associated with dietary diversity among studied children. Children in urban areas are 10% more likely to have a lower level of total DDS than those in rural areas (OR: 1.11 [0.79–1.56]; *p*-value: 0.5). Children whose mothers had a primary education level were 1.6-fold more likely to have a lower level of total DDS than children whose mothers had vocational training or college education (OR: 1.63 [0.68–3.92]; *p*-value: 0.28). Children from the poorest households were 1.2-fold more likely to have a lower DDS than those from the richest households (OR: 1.22 [0.79–1.87]; *p*-value: 0.37).

**Discussion:** A range of factors has been identified, contributing to the eating behaviors among CU5 in PNG, in which mother's education and household wealth are among the most important determinants of childhood dietary diversity as they have a direct effect on accessibility to and affordability of a variety of foods at the household level.

**Conclusion:** Evidence-based integrated and comprehensive approaches are needed to improve women education and household wealth, contributing to the improvement of food diversity among young children in PNG.

## Introduction

About one-third of the children do not achieve their full health and developmental potential due to inadequate dietary intake, and this has major health implications throughout life course (1, 2). According to the World Health Organization (WHO), malnutrition refers to deficiencies, excesses, or imbalances in a person's intake of energy and/or nutrients. Malnutrition can lead to two health conditions: (i) undernutrition, which includes stunting (low height for age), wasting (low weight for height), underweight (low weight for age), and micronutrient deficiencies or insufficiencies (a lack of important vitamins and minerals), and (ii) overweight and obesity, which are related to non-communicable diseases such as heart disease, stroke, diabetes, and cancer (3). Approximately 6.3 million children under 5 years of age (CU5) die every year globally, and about one-third of child deaths are particularly linked to diet-related health conditions (1, 4–6).

The World Health Assembly targets for 2025 and the United Nations' Sustainable Development Goals (SDGs) for 2030 have set out indicators of child malnutrition and for countries to monitor the food and nutrition status among CU5 (2, 4, 5). Nutrition is a cross-cutting issue and is vital for child health and development, but it is often neglected in the implementation of public health programs in low-middle-income countries (LMICs) (6). The recent changes in socioeconomic development in these countries are known to have greatly increased impacts on food choices and dietary habits among their populations (2, 7). In the Pacific Island Countries (PICs), including Papua New Guinea (PNG), food transition has been characterized by a move from traditional low-calorie diets to the consumption of processed high-calorie diets (8). At the same time, these countries show an emerging face of lifestyle diseases (9), with diet-related non-communicable diseases (NCDs) seen as an emerging public health problem, particularly among the adult population in urban areas (10, 11). By contrast, the problem of poor dietary intakes among child population continues to be a significant impediment in child health and development in these countries (12).

PNG is located just south of the equator and 160 km north of mainland Australia, in the South Pacific region, and consists of 22 provinces with a population of ~8.7 million in 2019. PNG has experienced dramatic social and economic changes following its opening to the outside world since the second half of the nineteenth century. The majority of the population (85%) lives in rural areas and is involved in subsistence-based agriculture (13). PNG has great potential to improve its socioeconomic development status through the economic development of its natural resources: land, agriculture, forestry, and fisheries (14). The mining and energy sectors contribute ~80% of the total export revenue of the country. The development of mining and extraction industries has led to significant social and economic changes across the country in recent years. With 38.2% of the population being under the age of 15 years, PNG has a young population (15).

Early childhood is a special developmental period offering great opportunity but also great vulnerability for individual health and development. Previous studies show that parental food choices, eating habits, and feeding strategies are the most important determinants affecting their children's eating behavior and nutritional diet, particularly at the early childhood (2, 16–20). Poor dietary intake, including breastfeeding and food supplementation constitutes larger risks contributing to childhood malnutrition in PNG (21–23), but there are few studies on diet-related child health issues in the food transition in PNG (24, 25).

**Figure 1:**
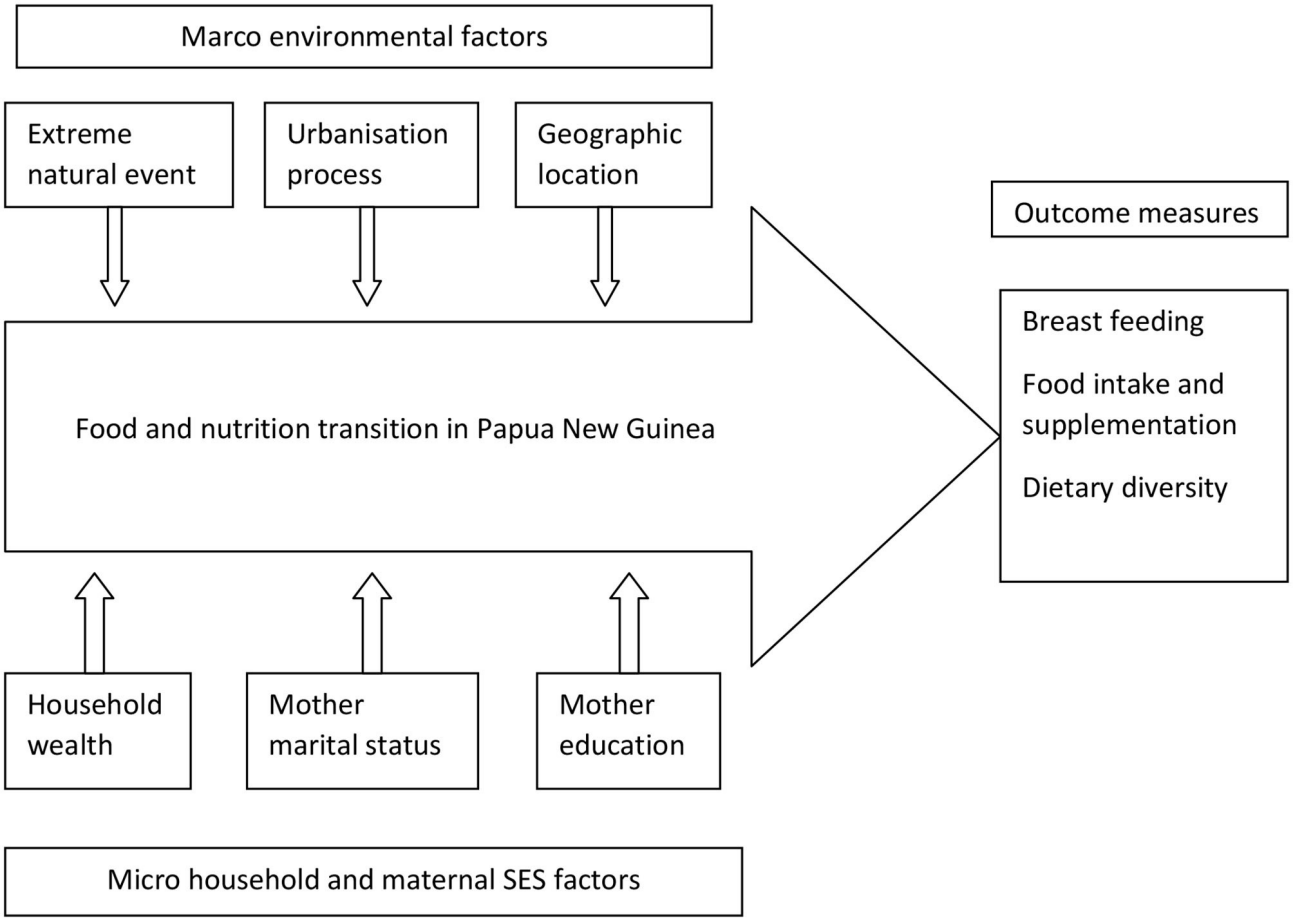
Conceptual framework for analyzing breastfeeding, food intake, and dietary diversity among children under 5 years old in PNG, PNGIMR's CHESS, 2020.

We hypothesize that the food and nutrition transition occurring in PNG posed increased health risks, particularly for child breastfeeding, food feeding, and dietary behaviors. Figure 1 showed the analytical conceptual framework for analyzing food transition in PNG, which may have been effected by macro environmental factors as well as micro household and maternal sociodemographic factors.

Macro environmental factors including extreme natural events such as drought, flood, earthquake, and landslide have direct impacts on agriculture production and distribution, hence affecting the transition at a large scale. Discussion of these factors will be covered in a separate paper. Urbanization processes redistribution of the population and social classes, contributing to socioeconomic development. The urbanization process is one of the important factors influencing availability and access to foods, especially in urban areas, hence contributing to the dietary diversity of urban children. Geographical location determines the local climate and culture, defining the food habits of a population. The four geographical regions of PNG with typical climate and ecosystems have various impacts on agriculture production, food processing, and eating behavior of the population, hence contributing to the nutritional status of children.

Micro environmental factors at the household and individual levels such as household socioeconomic status have a direct effect on a household's choice and consumption of food. Household wealth has direct influences on the food choice of household members and of children in particular. On the other hand, maternal demographic characteristics such as marital status and education could be also factors influencing breastfeeding practice, food supplementation, and dietary intake of children. Those children who are from households with better-off socioeconomic status and whose mothers have better education could have better dietary diversity and food intake than children from households with a poor socioeconomic status. The outcome of these processes is breastfeeding practice among women and food intake among children. All these contribute to the dietary diversity of the children, especially among CU5.

In this study, we attempted to report key indicators on eating behaviors among children aged 6–59 months, including breastfeeding, food supplementation, and dietary intake (16). We also explored the household and maternal sociodemographic factors influencing the children's dietary diversity in PNG.

## Methods

### Study Design, Location, and Population

**Figure 2:**
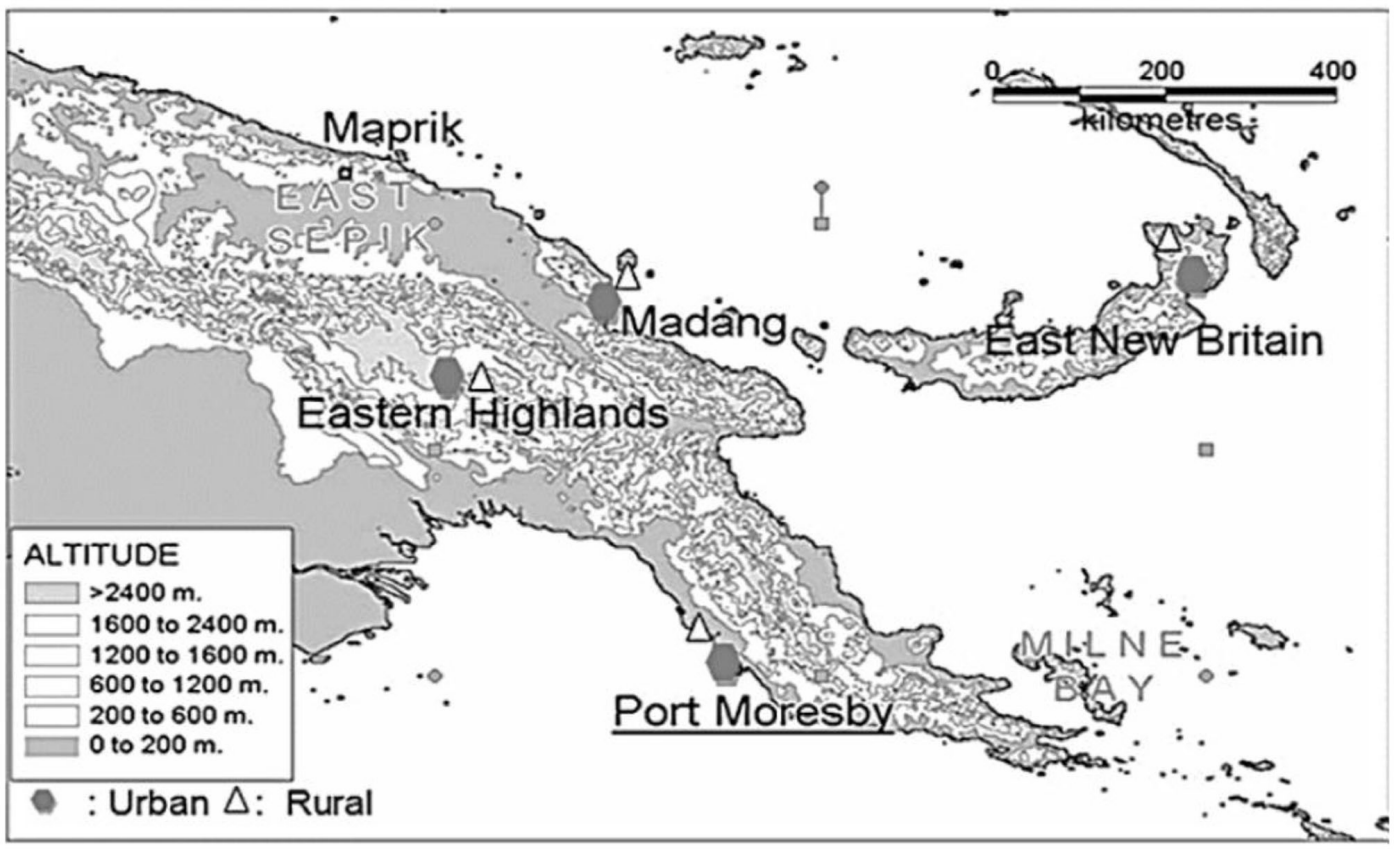
Geographic coverage and surveillance sites of PNGIMR's CHESS, 2020.

We used the data from the Comprehensive Health and Epidemiology Surveillance System (CHESS), operated by the Papua New Guinea Institute of Medical Research (PNGIMR) since 2010 (13, 17, 18). CHESS is designed as a cohort longitudinal follow-up study, and the CHESS methodology has been published elsewhere (19, 20). Figure 2 shows the five CHESS surveillance sites, located in four provinces representing the four geographic regions of PNG: Hiri and Port Moresby (POM) sites in Central Province for the southern region, Asaro and Goroka sites in Eastern Highlands Province (EHP) for the highlands region, the Newtown site in Madang Province for the Momase region, and Baining and Kokopo sites in East New Britain Province (ENB) for the islands region. These sites cover the population living in both urban and rural sectors, which are defined based on the PNG National Statistics Office's classifications and standards (26). Household interviews with parents and caregivers of child participants were conducted between July and December 2018 by CHESS national scientific officers, using a paper-based questionnaire, which is specifically designed for collecting information and data of CU5 who live in the surveillance sites.

To understand the dietary intake of children aged 6–59 months, parents or caregivers were asked questions on how many days they fed their children with a particular type of food in the typical week, meaning a normal week when the child diet was not affected by cultural, religious, or other social events. Mothers'/caregivers' responses were recorded with a score from 0 to 7, representing 0–7 days/week. The food items were categorized into nine groups of foods relevant to the PNG context: (i) root vegetables such as potato, sweet potato (kaukau), yam, and taro, providing starchy staples; (ii) green vegetables such as aibika and beans; (iii) fresh fruits such as papaw, orange, mango, and avocado; (iv) fresh meat such as chicken, pork, and fish; (v) tinned meat, fish, pork or chicken; (vi) fried foods which were cooked in the house such as fried flour and fried rice; (vii) fried foods purchased from shops such as flour balls, chips, and lamp flaps, providing a mixture of starch and meat; (viii) salt, providing additional iodine to children (all salt sold in PNG is iodized); and (ix) seasoning food such as Maggi and stock cube, providing additional flavor and taste.

### Variables

Variables on child background include age (in months), sex (male and female), residence (urban–rural), and province (surveillance sites). Key variables are derived mostly from the two data modules: (i) breastfeeding and nutrition and (ii) food intake and diet.

The CU5 dataset was linked with selected variables on household and maternal sociodemographic characteristic data, derived from the data component of the CHESS database comprising women of reproductive age, 15–49 years. The maternal variables included the highest educational attainment, employment, and marital status. These variables were linked with CU5 data using the unique household identification codes.

The household wealth index was calculated as an overall marker of household socioeconomic status. The estimates were based on the data on housing characteristics and household assets, extracted from the household data component of the CHESS database, using the factor analysis method. The household wealth indices were then divided into five categories (household wealth quintile), from the first to the fifth, representing the poorest, poorer, middle, richer, and richest groups of households, respectively. The process of calculating household wealth quintiles was discussed and published elsewhere (15).

### Data Analysis

Raw datasets were extracted in Microsoft Excel spreadsheet format, converted into Statistical Package of Social Science (SPSS) version 20.0 for data analysis. This process generated the results, and data were summarized as frequencies and percentages to understand the distributions of breastfeeding, eating behavior, and dietary intake and diversity among children aged 6–59 months.

**Figure 3:**
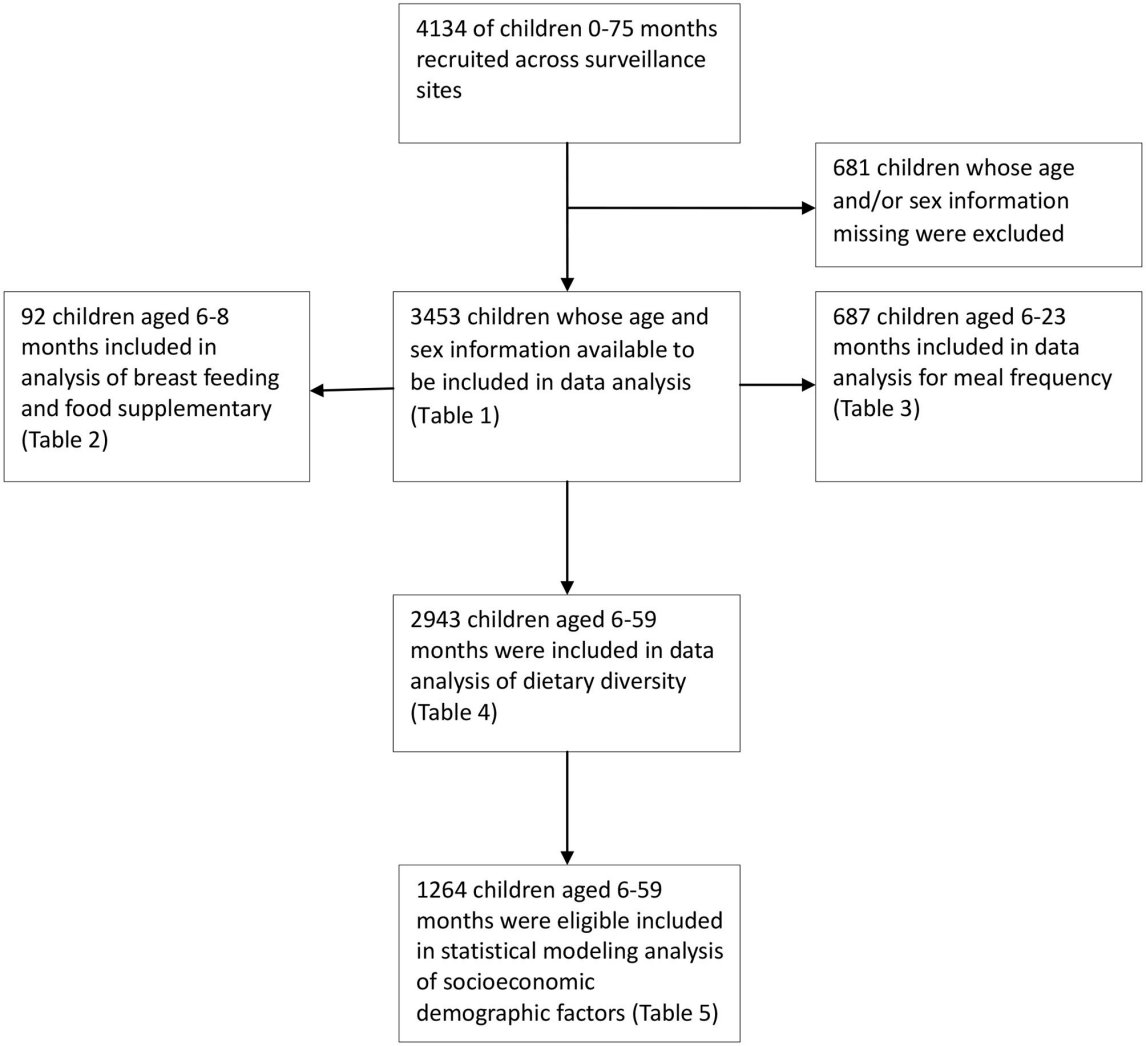
Flow diagram of child participation and selection for data analysis, PNGIMR's CHESS, 2020.

Figure 3 shows the flow diagram of child participation in the study and child selection for particular analysis. A total of 4,134 children aged 0–57 months were recruited across the surveillance sites, including 1,368 in urban areas (33%) and 2,766 in rural areas (67%). After sorting for children whose information on age and sex were missing, 3,458 children were found eligible and included in the data analyses for different purposes. The household and maternal socioeconomic and demographic characteristics of these children are presented in Table 1.

**Table 1:** Sociodemographic characteristics of all child participants and their mothers by surveillance site, PNGIMR's CHESS, 2020.

		**Central province**	**EHP**	**Madang province**	**ENB**	**POM**	**All sites**
Sector	Urban	0 (0.0%)	397 (34.5%)	331 (100.0%)	272 (31.0%)	372 (100.0%)	1,368 (33.1%)
	Rural	1,401 (100.0%)	755 (65.5%)	0 (0.00%)	606 (69.0%)	0 (0.0%)	2,766 (66.9%)
	Total	1,401 (100.0%)	1,152 (100.0%)	331 (100.0%)	878 (100.0%)	372 (100.00%)	4,134 (100.0%)
Age group in months	0–5	83 (6.8%)	25 (2.6%)	26 (8.4%)	36 (4.8%)	15 (6.9%)	185 (5.4%)
	6–11	70 (5.7%)	25 (2.6%)	27 (8.7%)	63 (8.5%)	16 (7.3%)	201 (5.8%)
	12–23	169 (13.8%)	124 (13.0%)	39 (12.6%)	131 (17.6%)	16 (7.3%)	479 (13.9%)
	24–35	287 (23.4%)	169 (17.7%)	59 (19.1%)	141 (18.9%)	59 (27.1%)	715 (20.7%)
	36–47	274 (22.3%)	197 (20.7%)	63 (20.4%)	147 (19.7%)	49 (22.5%)	730 (21.1%)
	48–59	342 (27.9%)	211 (22.1%)	71 (23.0%)	132 (17.7%)	40 (18.3%)	796 (23.1%)
	60–75	3 (0.2%)	202 (21.2%)	24 (7.8%)	95 (12.8%)	23 (10.6%)	347 (10.0%)
	Total	1,228 (100.0%)	953 (100.0%)	309 (100.0%)	745 (100.0%)	218 (100.0%)	3,453 (100.0%)
Sex of child	Male	629 (51.0%)	503 (52.3%)	158 (50.3%)	394 (52.8%)	111 (54.7%)	1,795 (51.9%)
	Female	604 (49.0%)	459 (47.7%)	156 (49.7%)	352 (47.2%)	92 (45.3%)	1,663 (48.1%)
	Total	1,233 (100.0%)	962 (100.0%)	314 (100.0%)	746 (100.0%)	203 (100.0%)	3,458 (100.0%)
Mother's educational attainment	Elementary	47 (6.2%)	41 (6.4%)	2 (0.6%)	27 (3.3%)	12 (6.3%)	129 (4.7%)
	Primary	584 (76.6%)	444 (69.4%)	114 (35.0%)	576 (70.7%)	142 (74.3%)	1,860 (68.0%)
	Lower secondary	112 (14.7%)	115 (18.0%)	170 (52.1%)	172 (21.1%)	29 (15.2%)	598 (21.9%)
	Upper secondary	12 (1.6%)	26 (4.1%)	26 (8.0%)	27 (3.3%)	3 (1.6%)	94 (3.4%)
	Vocational/college+	7 (0.9%)	14 (2.2%)	14 (4.3%)	13 (1.6%)	5 (2.6%)	53 (1.9%)
	Total	762 (100.0%)	640 (100.0%)	326 (100.0%)	815 (100.0%)	191 (100.0%)	2,734 (100.0%)
Mother marital status	In marriage	705 (56.0%)	483 (50.2%)	153 (46.6%)	386 (46.2%)	196 (58.5%)	1,923 (51.7%)
	In union	124 (9.9%)	176 (18.3%)	54 (16.5%)	84 (10.0%)	27 (8.1%)	465 (12.5%)
	Single mother	429 (34.1%)	303 (31.5%)	121 (36.9%)	366 (43.8%)	112 (33.4%)	1,331 (35.8%)
	Total	1,258 (100.0%)	962 (100.0%)	328 (100.0%)	836 (100.0%)	335 (100.0%)	3,719 (100.0%)
Household wealth quintile	Poorest	336 (23.9%)	404 (35.0%)	0 (0.0%)	27 (3.1%)	90 (24.2%)	857 (20.7%)
	Poorer	354 (25.2%)	259 (22.4%)	0 (0.0%)	139 (15.8%)	88 (23.7%)	840 (20.3%)
	Middle	274 (19.5%)	160 (13.9%)	3 (0.9%)	203 (23.1%)	83 (22.3%)	723 (17.5%)
	Richer	291 (20.7%)	228 (19.8%)	51 (15.4%)	278 (31.6%)	71 (19.1%)	919 (22.2%)
	Richest	148 (10.5%)	103 (8.9%)	277 (83.7%)	232 (26.4%)	40 (10.8%)	800 (19.3%)
	Total	1,403 (100.0%)	1,154 (100.0%)	331 (100.0%)	879 (100.0%)	372 (100.0%)	4,139 (100.0%)

Specifically, 92 children aged 6–8 months were included in the analysis of breastfeeding rate and food supplementation. It is recommended to introduce solid, semisolid, or soft foods to children in the age of 6–8 months as supplementary food to breastfeeding (2). For all children aged 6–8 months, they are recommended to be breastfed (up until 2 years old). These children are also recommended to start having supplementary foods, including soft, semisolid, and solid foods. It means all children in this age group should be both breastfed and fed with supplementary foods. There are three separate indicators to measure nutritional status among these children, namely (i) proportion of children having been currently breastfed (breastfeeding rate); (ii) proportion of children currently receiving supplementary foods; and (iii) proportion of children who are currently both breastfed and fed with supplementary foods. Analysis of drinks such as plain water, infant formula, milk, fruit juice, meat/vegetable water, vitamin/mineral supplements, oral rehydration solution (Oresol), and liquids (tea, soft drinks, and others) is not included in this paper. A chi-square test was used to compare proportions between child groups. Results are shown in Table 2.

**Table 2:** Breastfeeding and supplementary semi-solid and solid foods among infants aged 6–8 months, PNGIMR's CHESS, 2020.

		**No. of children aged 6–8 months**	**Currently being breastfed**	**Currently being fed with supplementary foods**	**Currently being breastfed and fed with supplementary foods**
Sector	Urban	28	20 (71.4%)	13 (46.4%)	9 (32.1%)
	Rural	64	58 (90.6%)	34 (53.1%)	32 (50.1%)
*p*-value		0.04	0.003	0.35	0.02
Province	Central	36	33 (91.7%)	23 (63.9%)	22 (61.1%)
	POM	10	5 (50.0%)	4 (40.0%)	2 (20.0%)
	EHP	10	9 (90.0%)	7 (70.0%)	7 (70.0%)
	Madang	10	7 (70.0%)	4 (40.0%)	2 (20.0%)
	ENB	26	24 (92.3%)	9 (34.6%)	8 (30.8%)
*p*-value		0.20	0.002	0.10	0.01
Sex	Male	48	40 (83.3%)	22 (45.8%)	18 (37.5%)
	Female	44	38 (86.4%)	25 (56.8%)	23 (52.3%)
*p*-value		0.35	0.59	0.29	0.36
	Total	92	78 (84.8%)	47 (51.1%)	41 (42.4%)

Furthermore, 687 children aged 6–23 months were included in the data analysis of frequency of meals for food supplementation. For children of the age group 6–23 months, they are recommended to be fed with supplementary foods with *at least three meals per day* for those children who are currently breastfed and *at least four meals per day* for those children who are currently not breastfed (27). Two indicators are calculated for reporting the food supplementation among children of this age group: (i) proportion of children who are currently breastfed and fed with three meals or more per day and (ii) proportion of children who are not currently breastfed but have four meals or more per day. Similar to the above analysis, the consumption of drinks is not analyzed in this paper. Chi-square tests were conducted to provide a significant level (*p*-value). Results are shown in Table 3.

**Table 3:** Frequency of meals among children aged 6–23 months who received solid, semisolid, and soft foods in the previous day, PNGIMR's CHESS, 2020.

		**No. of children aged 6–23 months**	**Currently breastfeeding and having 3+ meals per day**	**Currently not breastfeeding but having 4+ meals per day**
Sector	Urban	202	25 (12.4%)	8 (4.0%)
	Rural	485	118 (24.3%)	6 (1.2%)
*p*-value		0.03	0.45	0.92
Province	Central	243	52 (21.4%)	3 (1.2%)
	POM	34	2 (5.9%)	2 (5.9%)
	EHP	150	54 (36.0%)	3 (2.0%)
	Madang	66	8 (12.1%)	6 (9.1%)
	ENB	194	27 (13.9%)	0 (0.0%)
*p*-value		0.03	0.04	0.54
Sex	Male	362	80 (22.1%)	8 (2.2%)
	Female	325	62 (19.0%)	6 (1.8%)
*p*-value		0.01	0.54	0.53
	Total	687	142 (20.9%)	14 (2.1%)

Data of 2,943 children aged 6–59 months were analyzed for dietary intake and diversity. Results are shown in Table 4. Children aged 0–5 months were not included in the analysis as they should be exclusively breastfed while children aged 60 months and above were also not included because they are not defined as denominators in the measurement of dietary diversity among CU5. Dietary diversity score (DDS) was used as a proxy indicator to measure the variety of foods. This method is commonly used in low-resource settings (28, 29). There are different approaches to calculate DDS such as totaling the number of times when a child is fed with different types of food in a day (29). In this study, one score was given to the child, equivalent to 1 day, when the child was reported as having been fed with a particular type of food. The total DDS was calculated for each child by adding all the numbers of days when the child was fed with any food group mentioned above in a typical week prior to the interview. The mean of total DDS for a week was also calculated for each type of food as well as for all types of foods and disaggregated by province. Analysis of variance (ANOVA) and eta tests were used to compare means of total DDS between provinces. Results are shown in Table 4.

**Table 4:** Dietary intake and dietary diversity score among children aged 6–59 months for a typical week by surveillance site, PNGIMR's CHESS, 2020.

**Type of foods**	**Dietary diversity score**	**Central**	**EHP**	**Madang**	**ENB**	**POM**	**All sites**	**ANOVA *p*-value**	**Eta *p*-value**
Root vegetable	0	99 (8.6%)	20 (2.7%)	36 (13.7%)	74 (12.0%)	24 (13.0%)	253 (8.6%)		
	1	58 (5.1%)	5 (0.7%)	15 (5.7%)	64 (10.4%)	14 (7.6%)	156 (5.3%)		
	2	317 (27.6%)	50 (6.8%)	46 (17.6%)	93 (15.1%)	23 (12.4%)	529 (18.0%)		
	3	284 (24.8%)	98 (13.4%)	80 (30.5%)	88 (14.3%)	44 (23.8%)	594 (20.2%)		
	4	162 (14.1%)	118 (16.1%)	39 (14.9%)	62 (10.0%)	15 (8.1%)	396 (13.5%)		
	5	93 (8.1%)	62 (8.5%)	13 (5.0%)	50 (8.1%)	8 (4.3%)	226 (7.7%)		
	6	40 (3.5%)	53 (7.2%)	8 (3.1%)	7 (1.1%)	4 (2.2%)	112 (3.8%)		
	7	94 (8.2%)	323 (44.1%)	25 (9.5%)	149 (24.1%)	48 (25.9%)	639 (21.7%)		
	DK	0 (0.0%)	3 (0.4%)	0 (0.0%)	30 (4.9%)	5 (2.7%)	38 (1.3%)		
Mean DDS (SD)		2.89 (1.9)	5.1 (2.0)	2.8 (2.0)	3.5 (2.5)	3.5 (2.5)	3.7 (2.3)	<0.001	0.38
Green vegetable	0	232 (20.2%)	24 (3.3%)	25 (9.5%)	53 (8.6%)	31 (16.8%)	365 (12.4%)		
	1	213 (18.6%)	7 (1.0%)	6 (2.3%)	29 (4.7%)	17 (9.2%)	272 (9.2%)		
	2	338 (29.5%)	40 (5.5%)	40 (15.3%)	64 (10.4%)	19 (10.3%)	501 (17.0%)		
	3	196 (17.1%)	78 (10.7%)	49 (18.7%)	101 (16.4%)	41 (22.2%)	465 (15.8%)		
	4	90 (7.8%)	86 (11.7%)	31 (11.8%)	50 (8.1%)	15 (8.1%)	272 (9.2%)		
	5	43 (3.7%)	106 (14.5%)	14 (5.3%)	48 (7.8%)	13 (7.0%)	224 (7.6%)		
	6	8 (0.7%)	78 (10.7%)	27 (10.3%)	20 (3.2%)	5 (2.7%)	138 (4.7%)		
	7	25 (2.2%)	309 (42.2%)	70 (26.7%)	216 (35.0%)	38 (20.5%)	658 (22.4%)		
	DK	2 (0.2%)	4 (0.5%)	0 (0.0%)	36 (5.8%)	6 (3.2%)	48 (1.6%)		
Mean DDS (SD)		1.86 (1.6)	5.3 (2.0)	3.7 (2.5)	4.3 (2.5)	3.2 (2.5)	3.58 (2.5)	<0.001	0.56
Fruit	0	130 (11.3%)	34 (4.6%)	21 (8.0%)	32 (5.2%)	22 (11.9%)	239 (8.1%)		
	1	185 (16.1%)	81 (11.1%)	52 (19.8%)	65 (10.5%)	27 (14.6%)	410 (13.9%)		
	2	372 (32.4%)	202 (27.6%)	70 (26.7%)	108 (17.5%)	40 (21.6%)	792 (26.9%)		
	3	158 (13.8%)	151 (20.6%)	33 (12.6%)	149 (24.1%)	39 (21.1%)	530 (18.0%)		
	4	109 (9.5%)	103 (14.1%)	12 (4.6%)	61 (9.9%)	19 (10.3%)	304 (10.3%)		
	5	87 (7.6%)	32 (4.4%)	18 (6.9%)	56 (9.1%)	10 (5.4%)	203 (6.9%)		
	6	39 (3.4%)	25 (3.4%)	6 (2.3%)	19 (3.1%)	4 (2.2%)	93 (3.2%)		
	7	66 (5.8%)	94 (12.8%)	50 (19.1%)	95 (15.4%)	20 (10.8%)	325 (11.0%)		
	DK	1 (0.1%)	10 (1.4%)	0 (0.0%)	32 (5.2%)	4 (2.2%)	47 (1.6%)		
Mean DDS (SD)		2.42 (1.9)	3.25 (2.0)	2.84 (2.3)	3.36 (2.1)	2.63 (2.1)	2.90 (2.1)	<0.001	0.17
Fresh meat	0	56 (4.9%)	110 (15.0%)	29 (11.1%)	126 (20.4%)	17 (9.2%)	338 (11.5%)		
	1	60 (5.2%)	241 (32.9%)	90 (34.4%)	159 (25.8%)	20 (10.8%)	570 (19.4%)		
	2	191 (16.7%)	214 (29.2%)	33 (12.6%)	106 (17.2%)	19 (10.3%)	563 (19.1%)		
	3	229 (20.0%)	98 (13.4%)	45 (17.2%)	81 (13.1%)	48 (25.9%)	501 (17.0%)		
	4	248 (21.6%)	30 (4.1%)	26 (9.9%)	45 (7.3%)	23 (12.4%)	372 (12.6%)		
	5	165 (14.4%)	9 (1.2%)	12 (4.6%)	18 (2.9%)	27 (14.6%)	231 (7.8%)		
	6	51 (4.4%)	1 (0.1%)	2 (0.8%)	10 (1.6%)	3 (1.6%)	67 (2.3%)		
	7	144 (12.6%)	11 (1.5%)	24 (9.2%)	20 (3.2%)	23 (12.4%)	222 (7.5%)		
	DK	3 (0.3%)	18 (2.5%)	1 (0.4%)	52 (8.4%)	5 (2.7%)	79 (2.7%)		
Mean DDS (SD)		3.47 (2.0)	1.68 (1.3)	2.22 (2.0)	1.92 (1.8)	3.13 (2.1)	2.52 (2.0)	<0.001	0.46
Tinned meat	0	61 (5.3%)	53 (7.2%)	32 (12.2%)	75 (12.2%)	20 (10.8%)	241 (8.2%)		
	1	49 (4.3%)	105 (14.3%)	71 (27.1%)	86 (13.9%)	9 (4.9%)	320 (10.9%)		
	2	146 (12.7%)	150 (20.5%)	21 (8.0%)	79 (12.8%)	15 (8.1%)	411 (14.0%)		
	3	149 (13.0%)	156 (21.3%)	15 (5.7%)	100 (16.2%)	30 (16.2%)	450 (15.3%)		
	4	156 (13.6%)	102 (13.9%)	20 (7.6%)	80 (13.0%)	15 (8.1%)	373 (12.7%)		
	5	177 (15.4%)	75 (10.2%)	20 (7.6%)	67 (10.9%)	15 (8.1%)	354 (12.0%)		
	6	96 (8.4%)	31 (4.2%)	16 (6.1%)	24 (3.9%)	3 (1.6%)	170 (5.8%)		
	7	311 (27.1%)	55 (7.5%)	67 (25.6%)	54 (8.8%)	71 (38.4%	558 (19.0%)		
	DK	2 (0.2%)	5 (0.7%)	0 (0.0%)	52 (8.4%)	7 (3.8%)	66 (2.2%)		
Mean DDS (SD)		4.11 (2.3)	3.05 (1.9)	3.14 (2.7)	3.08 (2.2)	4.11 (2.6)	3.51 (2.3)	<0.001	0.29
Home-made fried foods	0	130 (11.3%)	210 (28.7%)	54 (20.6%)	138 (22.4%)	54 (29.2%)	586 (19.9%)		
	1	122 (10.6%)	137 (18.7%)	23 (8.8%)	135 (21.9%)	24 (13.0%)	441 (15.0%)		
	2	287 (25.0%)	144 (19.7%)	60 (22.9%)	78 (12.6%)	26 (14.1%)	595 (20.2%)		
	3	301 (26.2%)	117 (16.0%)	37 (14.1%)	77 (12.5%)	29 (15.7%)	561 (19.1%)		
	4	179 (15.6%)	46 (6.3%)	21 (8.0%)	47 (7.6%)	13 (7.0%)	306 (10.4%)		
	5	86 (7.5%)	30 (4.1%)	12 (4.6%)	40 (6.5%)	8 (4.3%)	176 (6.0%)		
	6	28 (2.4%)	7 (1.0%)	9 (3.4%)	15 (2.4%)	3 (1.6%)	62 (2.1%)		
	7	10 (0.9%)	8 (1.1%)	44 (16.8%)	23 (3.7%)	22 (11.9%)	107 (3.6%)		
	DK	4 (0.3%)	33 (4.5%)	2 (0.8%)	64 (10.4%)	6 (3.2%)	109 (3.7%)		
Mean DDS (SD)		2.44 (1.6)	1.72 (1.6)	2.71 (2.4)	2.18 (2.2)	2.28 (2.3)	2.20 (1.8)	<0.001	0.22
Shop-purchased fried foods	0	594 (51.8%)	142 (19.4%)	157 (59.9%)	220 (35.7%)	96 (51.9%)	1,209 (41.1%)		
	1	286 (24.9%)	134 (18.3%)	60 (22.9%)	185 (30.0%)	29 (15.7%)	694 (23.6%)		
	2	200 (17.4%)	165 (22.5%)	19 (7.3%)	60 (9.7%)	22 (11.9%)	466 (15.8%)		
	3	55 (4.8%)	120 (16.4%)	8 (3.1%)	42 (6.8%)	9 (4.9%)	234 (8.0%)		
	4	8 (0.7%)	87 (11.9%)	6 (2.3%)	16 (2.6%)	5 (2.7%)	122 (4.1%)		
	5	2 (0.2%)	33 (4.5%)	4 (1.5%)	6 (1.0%)	3 (1.6%)	48 (1.6%)		
	6	1 (0.1%)	11 (1.5%)	0 (0.0%)	1 (0.2%)	0 (0.0%)	13 (0.4%)		
	7	0 (0.0%)	17 (2.3%)	8 (3.1%)	4 (0.6%)	9 (4.9%)	38 (1.3%)		
	DK	1 (0.1%)	23 (3.1%)	0 (0.0%)	83 (13.5%)	12 (6.5%)	119 (4.0%)		
Mean DDS (SD)		0.73 (1.0)	2.08 (1.7)	0.78 (1.4)	1.07 (1.3)	1.04 (1.8)	1.20 (1.4)	<0.001	0.38
Stock cube^**^	0	295 (25.7%)	275 (37.6%)	230 (87.8%)	288 (46.7%)	71 (38.4%)	1,159 (39.4%)		
	1	180 (15.7%)	100 (13.7%)	9 (3.4%)	91 (14.7%)	2 (1.1%)	382 (13.0%)		
	2	165 (14.4%)	138 (18.9%)	5 (1.9%)	31 (5.0%)	8 (4.3%)	347 (11.8%)		
	3	166 (14.5%)	82 (11.2%)	5 (1.9%)	37 (6.0%)	16 (8.6%)	306 (10.4%)		
	4	148 (12.9%)	50 (6.8%)	0 (0.0%)	18 (2.9%)	8 (4.3%)	224 (7.6%)		
	5	95 (8.3%)	31 (4.2%)	4 (1.5%)	13 (2.1%)	15 (8.1%)	158 (5.4%)		
	6	18 (1.6%)	9 (1.2%)	1 (0.4%)	19 (3.1%)	1 (0.5%)	48 (1.6%)		
	7	70 (6.1%)	14 (1.9%)	8 (3.1%)	65 (10.5%)	49 (26.5%)	206 (7.0%)		
	DK	10 (0.9%)	33 (4.5%)	0 (0.0%)	55 (8.9%)	15 (8.1%)	113 (3.8%)		
Mean DDS (SD)		2.19 (2.1)	1.6 (1.7)	0.39 (1.3)	1.81 (2.5)	2.85 (3.0)	1.82 (2.2)	<0.001	0.28
Salt^*^	0	357 (31.1%)	304 (41.5%)	205 (78.2%)	273 (44.2%)	51 (27.6%)	1,190 (40.4%)		
	1	200 (17.4%)	78 (10.7%)	7 (2.7%)	61 (9.9%)	9 (4.9%)	355 (12.1%)		
	2	164 (14.3%)	85 (11.6%)	4 (1.5%)	16 (2.6%)	11 (5.9%)	280 (9.5%)		
	3	68 (5.9%)	49 (6.7%)	1 (0.4%)	22 (3.6%)	11 (5.9%)	151 (5.1%)		
	4	52 (4.5%)	71 (9.7%)	0 (0.0%)	5 (0.8%)	9 (4.9%)	137 (4.7%)		
	5	42 (3.7%)	35 (4.8%)	3 (1.1%)	14 (2.3%)	7 (3.8%)	101 (3.4%)		
	6	20 (1.7%)	21 (2.9%)	1 (0.4%)	7 (1.1%)	0 (0.0%)	49 (1.7%)		
	7	225 (19.6%)	41 (5.6%)	39 (14.9%)	168 (27.2%)	78 (42.2%)	551 (18.7%)		
	DK	19 (1.7%)	48 (6.6%)	2 (0.8%)	51 (8.3%)	9 (4.9%)	129 (4.4%)		
Mean DDS (SD)		2.35 (2.6)	1.96 (2.2)	1.05 (2.4)	2.64 (3.1)	3.58 (3.1)	2.26 (2.7)	<0.001	0.22
Total of children		1,147 (100.0%)	732 (100.0%)	262 (100.0%)	617 (100.0%)	185 (100.0%)	2,943 (100.0%)		
Mean total DDS (SD)		22.49 (11.45)	25.31 (9.3)	19.65 (12.59)	23.06 (14.42)	26.23 (13.56)	23.30 (11.96)	<0.001	0.14

* *Any type of salt, including iodized salt was put directly on any food fed to the child participants*.

** *Refers to food seasoning that adds tastes and flavor and put directly on any food fed to child participants*.

*DK, do not know*.

Lastly, the data of 1,264 children aged 6–59 months were found eligible and included in statistical modeling for the analysis of the association of household and maternal socioeconomic factors with dietary diversity among these children. Multivariate logistic regression (MLR) analysis was conducted for this purpose. The mean of total DDS was used as the cutoff point to divide children into two groups: children with low total DDS (below the mean) and those with high total DDS (above the mean). A new categorical variable on total DDS level (low and high) was created and included in the model MLR as a dependent variable, with low total DDS level being used as a reference category. Independent variables, including household and maternal socioeconomic and demographic variables, were included in the MLR model as factors to predict the outcome of a low-level total DDS. Non-significant variables were excluded from the models, and only significant factors remained, including resident location (urban–rural sector), province, sex of the child, child age group, mother's highest educational attainment (elementary, primary, lower secondary, upper secondary, and vocational training and above), mother's marital status (currently married/in union, not married/in union, and single mother), child age group (6–11, 12–23, 24–35, 36–47, and 48–59 months), sex of the child (male or female), and household wealth quintile (from poorest to richest), assuming other predictors remain constant. The model of the main effect was selected to produce maximum likelihood estimates of odds ratios (ORs) of having low total DDS. A statistical likelihood test was also selected in the model to provide estimates of 95% CI of OR and significance levels. Results are shown in Table 5.

**Table 5:** Odds ratio of having low level of total dietary diversity scores among children aged 6–59 months by household and maternal socioeconomic demographic factors in PNG, PNGIMR's CHESS, 2020.

**Factor**	**Category**	**No. of children**	**%**	**Odds ratio**	**Lower bound**	**Upper bound**	***p*-value**
Sector	Urban	300	23.7%	1.11	0.79	1.56	0.54
	Rural	964	76.3%	Ref.			
Province	Central	556	44.0%	0.72	0.40	1.31	0.28
	EHP	384	30.4%	1.16	0.67	2.02	0.59
	ENB	246	19.5%	0.59	0.33	1.05	0.07
	POM	78	6.2%	Ref.			
Sex of child	Male	668	52.8%	0.99	0.79	1.23	0.90
	Female	596	47.2%	Ref.			
Child age group	6–11	91	7.2%	1.11	0.69	1.79	0.66
	12–23	196	15.5%	1.07	0.75	1.53	0.70
	24–35	312	24.7%	0.89	0.65	1.21	0.47
	36–47	305	24.1%	0.98	0.72	1.35	0.92
	48–59	360	28.5%	Ref.			
Mother education	Elementary	77	6.1%	2.30	0.85	6.20	0.10
	Primary	942	74.5%	1.63	0.68	3.92	0.28
	Lower secondary	196	15.5%	2.00	0.80	4.98	0.14
	Upper secondary	27	2.1%	2.80	0.86	9.14	0.09
	Vocational/college+	22	1.7%	Ref.			
Mother marital status	Married	543	43.0%	0.94	0.74	1.20	0.62
	In union	170	13.4%	0.88	0.62	1.25	0.47
	Single mother	551	43.6%	Ref.			
HH wealth quintile	Poorest	251	19.9%	1.22	0.79	1.87	0.37
	Poorer	285	22.5%	0.97	0.64	1.45	0.87
	Middle	264	20.9%	1.07	0.71	1.62	0.74
	Richer	308	24.4%	1.06	0.71	1.57	0.77
	Richest	156	12.3%	Ref.			
Total of children		1,264	100.0%				

*Multivariate logistic regression model with a dependent variable of total DDS level with low total DDS (below the mean) as a reference category. HH and maternal factors included in the model were urban–rural sector; province; sex and age group of child; mother's age group, educational attainment, occupation, and marital status; and household wealth quintile*.

## Results

### Household, Maternal, and Child Socioeconomic Demographic Characteristics

Table 1 shows the sociodemographic characteristics of all child participants and their mother. About 66.9% of child participants were from the rural areas and 33.1% from the urban areas. Note that all children in the Central Province were from the rural sector, but all children in Madang Province and POM were from the urban sector. The proportion of children increased with the increase in age group, from 5.4% in the age group 0–5 to 23.1% in the age group 48–59 months. About 51.9% were males, and 48.1% were females. In regard to maternal characteristics, 68.0% of mothers attained primary education, compared to 21.9, 3.4, and 1.9% who attained lower, upper secondary, and vocational/college education, respectively. Marital status shows that 51.7% were married, and 12.5% were in union, but 35.8% reported as not being married or in union.

### Breastfeeding and Supplementary Food

As shown in Table 2, about 85% children were currently being breastfed, and more than 50% were given solid, semisolid, or soft foods. The proportion of children aged 6–8 months being breastfed and given supplementary food (solid and semisolid foods) was about 42%, with a higher rate in rural areas (47%) than in urban areas (32%). The higher rate of breastfeeding and appropriate food supplementation was reported in EHP and Central Province, about 60%. The proportion of female children being breastfed and given supplementary foods was significantly higher than male counterparts, 50 and 35%, respectively (chi-square test results <0.5).

### Meal Frequency

Table 3 shows that above 20% of children aged 6–23 months were currently breastfed, plus received solid, semisolid, and soft foods three times per day or more, with a lower proportion in urban (12%) than in rural areas (24%). EHP recorded the highest proportion (36%) followed by Central Province (21%), but the lowest proportion was in POM (6%). Among children who were currently not breastfed, only 14 children (2%) received solid, semisolid, and soft foods or milk feeds four times per day or more.

### Dietary Intake

As shown in Table 4, child participants were reportedly being fed with root vegetables for 3.7 days/week on average, with the highest rate reported in EHP (5.1 days/week), but the lowest in Madang Province (2.8 days/week). Similarly, green vegetables were being fed to children with the highest rate in EHP (5.3 days/week) and the lowest in Central Province (1.8 days/week). Fresh meat or fish were given to children for 2.52 days/week, compared to 3.51 days/week for tinned meat or tinned fish. Children were reportedly being fed with homemade fried foods with the highest rate reported in Madang Province (2.7 days/week) and the lowest rate in EHP (1.7 days/week). In contrast, children were fed with purchased fried foods with the highest rate in EHP (2.08 days/week) and the lowest in Central Province (0.73 days/week). Stock cube directly put on the food was reported, with the highest rate in POM (2.85 days/week) and the lowest in Madang Province (0.39 days/week). Similarly, salt was put directly on the food for children, with the highest rate reported in POM (3.5 days/week) and the lowest rate in Madang Province (1.05 days/week).

### Dietary Diversity Score

The mean of total DDS among children was 23.3 (SD: ±11.96) for all children across the sites. The mean DDS varied across the sites, with the most diversity found in POM (26.23), followed by EHP (25.31), ENB (23.06), and Central Province (22.49), and the lowest in Madang Province (19.65). Both ANOVA and eta tests showed that the differences between means of DDS were statistically significant between provinces across different types of foods and for all types of foods.

**Figure 4:**
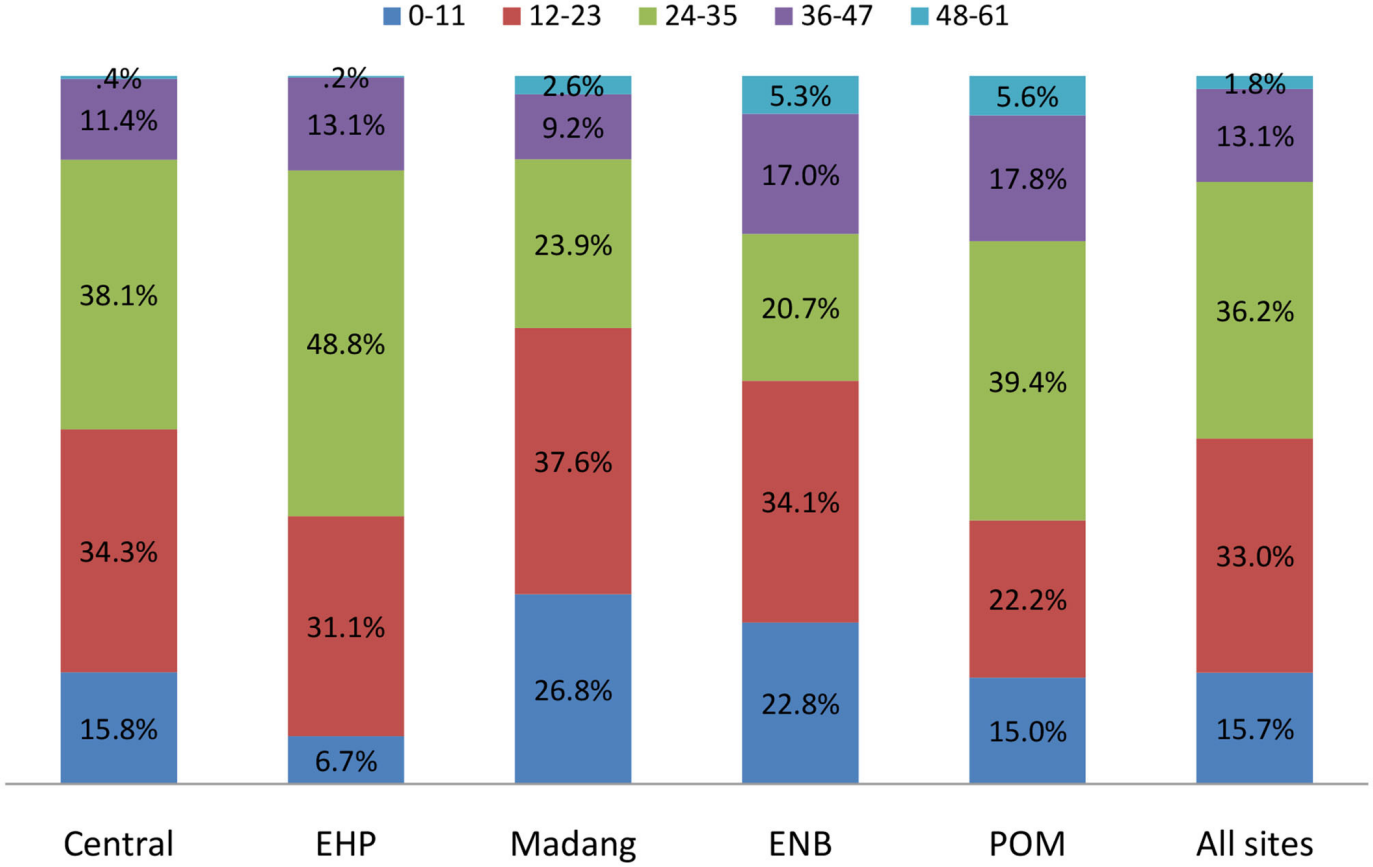
Distribution of child participants by total dietary diversity scores by province, PNGIMR's CHESS, 2020.

Furthermore, Figure 4 represents the distribution of CU5 by total DDS in each province. The data revealed that about 49% of children in all sites had a total DDS below the mean of 23.3 scores. We used the value of 23 scores as the cutoff point to divide child participants into two groups: low DDS (≤23) and high DDS (≥24). EHP had the highest proportion of children (49%) with a high total DDS (24–35 points) in a typical week preceding the interview, followed by POM (40%) and Central Province (38%). On the other hand, Madang, Central, and ENB provinces had the highest proportions of children with a low total DDS (12–23 points), ranging 38–34%.

### Household, Maternal, and Child Sociodemographic Factors Associated With DDS

In this section, we looked at the socioeconomic determinants of total DDS among children aged 6–59 months. Table 5 presents the estimated OR with 95% CI and *p*-value, using the MLR model (no data of children in Madang Province were included because of either missing or ineligible values in mother's education, marital status, or household wealth quintile). Results showed that children in urban areas are more likely to have a low level of total DDS than children in rural areas (OR: 1.11 [0.79–1.56]; *p*-value: 0.5). Similarly, children in EHP are more likely to have a low DDS than children in POM (OR: 1.16 [0.67–2.02]; *p*-vale: 0.28). No significant difference was observed between male and female children and across child age groups (except children of age group 24–35 months).

Among maternal sociodemographic variables, mother's highest educational level attainment has significant associations with the total DDS across all educational levels. Specifically, children of women with lower secondary education were more likely to have a low total DDS than children from mothers with vocational training or college education and above (OR: 2.00 [0.8–4.98]; *p*-value: 0.14). Children whose mothers lived in union were less likely to have a low DDS than children of single mothers (OR: 0.88 [0.62–1.25]; *p*-value: 0.47). Noticeably, children from the poorest quintile were more likely to have a low total DDS than those from the richest quintile (OR: 1.22 [0.79–1.87]; *p*-value: 0.37).

## Discussion

This study has provided large data collected from both rural and urban areas in five main provinces representing four geographic regions of PNG and covered a range of information on eating behaviors among children aged 6–59 months. Household and maternal socioeconomic demographic data were collected, facilitating analyses of factors associated with the choice of food in PNG.

### Eating Behavior and Associated Factors Among CU5 in PNG

More than half of the breastfed children aged 6–8 months were given supplementary foods, but with appropriate introduction of solid and semisolid foods in less than half of the children. For children aged 6–23 months, 20% of currently breastfed children meet the minimum meal frequency requirement, compared to only 2% of currently non-breastfed children. These proportions are lower than those found in a previous study (27), but this suggests that there is a need to further strengthen the nutrition education for mothers on the recommended child feeding practices.

Most children aged 6–59 months were fed with vegetables and fruits, with dietary variations. Child participants in the urban sites of POM and Madang Province, with diet low in vegetables and fruits, not only reflect limited subsistence farming and limited availability of and accessibility to these foods but also family socioeconomic factors and dietary choices in the urban areas. High consumption of vegetables in the EHP reflects subsistence farming and availability of and accessibility of these foods through its own production or market. The households in EHP may have limited access to these foods in dry season, often in the third quarter, but also due to social instability associated with the national election in 2017.

Children of this age group were more likely to be fed fresh and tinned meat or fish, especially in POM, which may be due to the availability of and accessibility to this food, but also the food preferences of the parents living in the urban areas. This is likely associated with household affordability and convenience. Fresh meat is more expensive than tinned meat, but tinned meat can be kept for a long time without a fridge or freezer. This is particularly true in the context of PNG, where 20% of households had access to electricity and 5% owned a fridge or freezer (15). Tinned meat, fish, rice, and flour, which are either locally made or imported, are available in village food stores all over the country, while fresh fish are more likely available in coastal areas such as Madang Province and ENB. Access to these foods is therefore more related to household socioeconomic status (30). Fresh and tinned meat and fish are much higher in protein, zinc, and energy than the local root vegetables such as sweet potatoes, yam, and taros. Dietary effects on the linear growth of children were discussed by Smith et al. (31). A previous study in PNG suggests that high consumption of local root vegetables (with the exception of potatoes) was correlated with reduced growth of children and that protein and/or zinc limitation might be a major component of slow growth in PNG, though growth is usually limited by multiple, simultaneous deficiencies (32). Unusually for PNG, the protein content of bush meat is an important part of the diets and exceeds the recommended Food and Agriculture Organization (FAO)/WHO levels (33).

These children were more likely to be fed homemade rather than purchased fried food, especially in Central Province, POM, and Madang Province, indicating the common practice in urban settings and rural areas which have undergone an urbanization process. Mothers from better-off families could be more concerned about the quality of fried food sold in the shop, so they make it at home for their children. But the higher proportion of children in EHP who consumed purchased fried foods may suggest their availability and affordability and the food choices and preferences by adults with children. Cash income from subsistence cash cropping, particularly from coffee after July when the data collection was conducted, may explain the situation (34). The consumption of fried foods with high fat intake raises child health issue and also public health concern as individuals may be at a greater risk of developing heart disease, diabetes, obesity, and cancer (35, 36). Access to and uptake of iodized salt can alleviate iodine deficiency and improve health outcomes (37). Mothers from poorer households are likely to prefer salt, while mothers from better-off households are more likely to prefer stock cubes. That is because, in PNG, salt is cheaper and can be used for more days than stock cubes. However, the higher proportion of children in Central Province, POM, and ENB consuming both salt and stock cubes raises child health concern and also poses great health risks for adults. There is a relationship between increased salt consumption and subsequent risk of cardiovascular diseases. Children with high salt levels are at risk of raised blood pressure and become adult with elevated blood pressure, which increases the risk of kidney disease, heart disease, heart attack, and stroke. High salt intake also promotes overconsumption of fatty foods, which increases the risk of obesity. By contrast, lower sodium intake could also have negative impact on child health and development (38).

### Policy and Program Implications

Our study shows that children from wealthier households have better dietary diversity than children from poor households. This could be because mothers from better-off households often purchase more foods and feed their children with more variety of foods (34, 36, 39). In PNG, food supply relies more on agricultural production at the household level rather than the food chain systems (40). Previous studies in Ethiopia suggest that household livelihood strategies with appropriate family farming interventions can improve household income and productivity. Furthermore, each additional food group produced by the household increases the food quantity and diversity for children (27, 39, 41). Recent analyses in Bangladesh found household income to be a significant determinant of household dietary diversity (28, 37). In PNG, household wealth can also be improved by increasing the transportation and circulation of agricultural products across provinces and geographical regions. PNG women's participation in agriculture works and other livelihood activities likely constrained their time for child care, especially for single mothers. Hence, PNG men are encouraged to support women in taking care of children so that women could optimally breastfeed and provide food supplements for their children (37).

Our study revealed that mother's education is a significant factor contributing to the higher level of dietary diversity among children. This finding is consistent with a previous study suggesting that women's higher education is a protective factor against childhood malnutrition. More highly educated mothers are more likely to have better knowledge and practices of nutritious food habits. Nutritional education programs can improve breastfeeding practices and dietary diversity. These results are supported by findings from other developing countries (42). However, the translation of education to improved dietary practices and subsequent improvements in child nutritional status could be a lengthy process in PNG.

Building human resource capacity to address nutritional issues is crucial in PNG, requiring strategy, consistent financing, and leadership to successfully implement the National Nutritional Strategy 2015–2020 (33). Stand-alone nutrition education programs have been carried out in the Pacific region and PNG in particular (8, 40). Nutritional education should be integrated into postnatal care services and become standard practice at primary health facilities in PNG. Health workers should be trained in delivering messages on how to improve nutrition outcomes among children. Research findings are needed to support interventions to improve healthy eating behavior, food choices among household members and young women in particular, to ensure that nutritional education reaches population living in rural and remote areas. Home-based nutrition counseling would improve child dietary intake, in both quantity and quality, contributing to the improvement of nutrition status of PNG children in the long term.

### Data Limitation

Data used in this study have some limitations. First, the study was conducted with CU5 participants living within the surveillance sites of PNG. Hence, the data are not nationally representative. Second, parents and caregivers reported on breastfeeding and food feeding in a typical week, but the provided information might have resulted in recall biases, and it may not be representative; for instance, food diversity varies across seasons in a year. Third, the DDS method is among the few research methods available to measure food and dietary diversity at the population level. This method appears to have some limitations when we applied it to our study. The same scoring system was applied to all child age groups and across all types of foods with one score being given to a child whenever the child was reported to be fed with a type of food regardless of the amount of food being given. In the local context of PNG, a variety of foods are given to children that may be not necessarily be limited to the nine groups, as defined in this study. The DDS method is for first time applied to assess food diversity among CU5 in PNG; hence, a comparison between our findings and data from previous studies in PNG or similar settings is relatively limited.

## Conclusion

Our study confirms that the urban–rural sector, household wealth, and maternal education attainment are the key socioeconomic determinants of dietary diversity and intake among CU5 in PNG. Evidence-based interventions are needed to improve dietary diversity and intake among young children. That could reduce the burden of diet-related morbidity and mortality in PNG in the future. Any solution aiming at improving health and nutrition among PNG children needs to address the fundamental economic issues at the household level. There is a need of an evidence-based and location-specific approach, considering the differentials in household and maternal socioeconomic characteristics. Monitoring and reporting breastfeeding, food supplementation, and dietary intake not only provide information about the health status of children but also inform policy for effectively programming interventions at the national and local levels. We call for an integrated comprehensive approach to improving household wealth, contributing to the improvement of the nutritional status of children in PNG.

## Data Availability Statement

The raw data supporting the conclusions of this article will be made available by the authors, without undue reservation.

## Ethics Statement

The studies involving human participants were reviewed and approved by the CHESS was granted ethics approvals from Internal Review Board of PNG Institute of Medical Research (IRB's Approval no. 18.05) and the Medical Research Advisory Committee of Papua New Guinea (MRAC's Approval no. 18.06). These approvals covered all the data components under the CHESS, including data of children under 5 years of age, which were used in this manuscript. Informed consent was sought from self-identified household heads and woman participants. Women were informed about their right to withdraw from the study at any stage. Written informed consent to participate in this study was provided by the participants' legal guardian/next of kin.

## Author Contributions

BP designed the CHESS, conceptualized the paper, analyzed and interpreted the data, drafted, revised, finalized, and submitted the manuscript. VS supervised the fieldwork, collected data, and provided the inputs. AO reviewed and commented and edited the manuscript. WP oversaw the CHESS and approved the submission of the manuscript. All authors contributed to the article and approved the submitted version.

## Conflict of Interest

The authors declare that the research was conducted in the absence of any commercial or financial relationships that could be construed as a potential conflict of interest.
